# Artificial Intelligence in Endoscopic Ultrasound for Pancreatic Cancer: Where Are We Now and What Does the Future Entail?

**DOI:** 10.3390/jcm11247476

**Published:** 2022-12-16

**Authors:** Dushyant Singh Dahiya, Mohammad Al-Haddad, Saurabh Chandan, Manesh Kumar Gangwani, Muhammad Aziz, Babu P. Mohan, Daryl Ramai, Andrew Canakis, Jay Bapaye, Neil Sharma

**Affiliations:** 1 Department of Internal Medicine, Central Michigan University College of Medicine, Saginaw, MI 48601, USA; 2 Division of Gastroenterology and Hepatology, Indiana University School of Medicine, Indianapolis, IN 46202, USA; 3 Division of Gastroenterology and Hepatology, CHI Creighton University Medical Center, Omaha, NE 68131, USA; 4 Department of Internal Medicine, The University of Toledo Medical Center, Toledo, OH 43614, USA; 5 Department of Gastroenterology, The University of Toledo Medical Center, Toledo, OH 43614, USA; 6 Division of Gastroenterology and Hepatology, University of Utah School of Medicine, Salt Lake City, UT 84132, USA; 7 Division of Gastroenterology and Hepatology, University of Maryland School of Medicine, Baltimore, MD 21201, USA; 8 Department of Internal Medicine, Rochester General Hospital, Rochester, NY 14621, USA; 9 Parkview Cancer Institute, Fort Wayne, IN 46845, USA; 10 Interventional Oncology & Surgical Endoscopy Programs (IOSE), Parkview Health, Fort Wayne, IN 46845, USA

**Keywords:** artificial intelligence, endoscopic ultrasound, pancreatic cancer, chronic pancreatitis, autoimmune pancreatitis

## Abstract

Pancreatic cancer is a highly lethal disease associated with significant morbidity and mortality. In the United States (US), the overall 5-year relative survival rate for pancreatic cancer during the 2012–2018 period was 11.5%. However, the cancer stage at diagnosis strongly influences relative survival in these patients. Per the National Cancer Institute (NCI) statistics for 2012–2018, the 5-year relative survival rate for patients with localized disease was 43.9%, while it was 3.1% for patients with distant metastasis. The poor survival rates are primarily due to the late development of clinical signs and symptoms. Hence, early diagnosis is critical in improving treatment outcomes. In recent years, artificial intelligence (AI) has gained immense popularity in gastroenterology. AI-assisted endoscopic ultrasound (EUS) models have been touted as a breakthrough in the early detection of pancreatic cancer. These models may also accurately differentiate pancreatic cancer from chronic pancreatitis and autoimmune pancreatitis, which mimics pancreatic cancer on radiological imaging. In this review, we detail the application of AI-assisted EUS models for pancreatic cancer detection. We also highlight the utility of AI-assisted EUS models in differentiating pancreatic cancer from radiological mimickers. Furthermore, we discuss the current limitations and future applications of AI technology in EUS for pancreatic cancers.

## 1. Introduction

Pancreatic cancer has been identified as the seventh leading cause of cancer-related death worldwide [1]. Pancreatic ductal adenocarcinoma (PDAC), an invasive mucin-producing neoplasm with an intense stromal desmoplastic reaction, is the most common (90%) subtype of pancreatic cancer [2,3]. The median age at diagnosis for pancreatic cancer is about 70 years. PDAC is slightly more common in males compared to females on a global scale (age-standardized incidence rate of 5.5 per 100,000 in men vs. 4 per 100,000 in women) [4,5]. Current estimates by the National Cancer Institute (NCI) predict 62,210 new cases of pancreatic cancer in the US in 2022, representing 3.2% of all new cancer diagnoses [6].

Most patients with pancreatic cancer lack obvious clinical signs and symptoms until they have advanced-stage disease. Furthermore, traditional imaging techniques such as computer tomography (CT) and magnetic resonance imaging (MRI) may not be able to detect small or premalignant pancreatic lesions. Therefore, an early diagnosis is often difficult to establish. Hence, due to a late initial presentation, patients often have advanced-stage disease with widespread metastasis, leading to poor clinical outcomes and high mortality rates [3,7]. In the US, the age-adjusted death rate for pancreatic cancer was noted to be 11.1 per 100,000 men and women per year between 2015–2019 [6]. However, it is worth noting that the 5-year relative survival rate for pancreatic cancer in the US has continued to rise from 3.2% in the 1970s to 11.5% for the 2012–2018 period, reflecting possible improvements in diagnostic and management strategies [6].

Endoscopic ultrasound (EUS) has the greatest specificity and sensitivity for the diagnosis of pancreatic lesions and, in particular, pancreatic cancer. Recently, biopsies via EUS have shifted from fine-needle aspiration (FNA) to fine-needle biopsy (FNB). EUS combined with FNB has a specificity and sensitivity greater than 90% for the detection of pancreatic cancer [8,9]. However, EUS does not have widespread availability and utilization due to the need for additional training, a steep learning curve, operator dependence, the cost of equipment, and the need for sedation.

Over the years, gastroenterologists have typically relied on individualized manual analysis and the interpretation of EUS and cross-sectional radiographic images to diagnose, classify, and plan interventions for patients with gastrointestinal (GI) neoplasms [10]. This has inevitably led to significant variability in diagnosis based on clinical proficiency, expertise, and individual bias. However, recently, AI has gained immense popularity in GI, particularly for luminal and pancreaticobiliary disorders, due to its ability to analyze large sets of data with a high degree of accuracy [11]. AI algorithms not only assist with the rapid diagnosis of GI neoplasms but also reduce inter-observer variability, decrease rates of misdiagnosis, and standardize the interpretation of radiological and histopathological images, leading to accurate diagnosis and improvements in clinical outcomes [10,11,12].

EUS is the imaging modality of choice for pancreatic cancers and is preferred over conventional CT scans and MRIs due to its high diagnostic yield and negative predictive value [13]. In current literature, numerous AI models have been successfully integrated with EUS [14]. This has led to the early detection of pancreatic cancer, thereby expediting management, reducing the risk of mortality, and decreasing the overall healthcare burden on individuals and healthcare systems across the globe [15,16]. In this comprehensive review, we focus our discussion on AI and its application in EUS for the detection and differentiation of pancreatic neoplasms from other disease entities such as chronic pancreatitis (CP) and autoimmune pancreatitis (AIP). Furthermore, we also highlight the limitations and future applications of AI technology in EUS for pancreatic cancers.

## 2. Discussion

### 2.1. Artificial Intelligence and Its Utility in Gastroenterology

**Figure 1:**
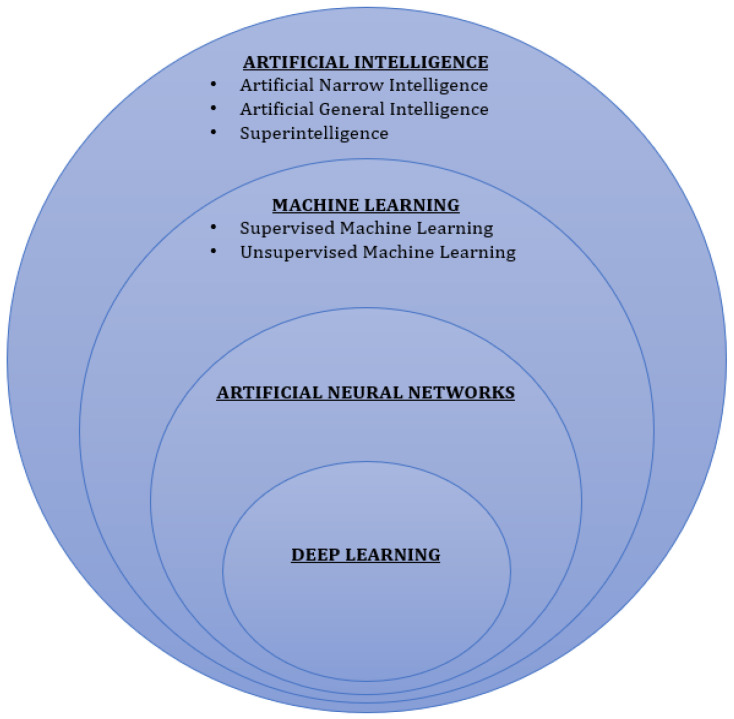
Types of artificial intelligence.

AI is a highly complex integration of computer systems and software to design computer algorithms that display the properties of critical thinking and intelligence (Figure 1) [12]. In a broader sense of the term, AI aims to replicate human intelligence with learning abilities and complex problem-solving skills. Since it was first described by John McCarthy in 1956, AI algorithms have undergone a major transformation from artificial narrow intelligence (ANI), which was primarily designed to perform simple pre-determined tasks, to artificial general intelligence (AGI) and superintelligence, which can analyze large quantities of data and solve complex problems accurately [17,18]. The three major branches of AI that are slowly revolutionizing clinical practice include machine learning (ML), artificial neural networks (ANNs), and expert systems (ES).

ML is a branch of AI that allows software applications to attain efficiency in predicting outcomes of interest without explicit programming, using already available historical data as input [19]. It can be further subdivided into supervised and unsupervised learning. Supervised ML provides data in the form of input–output pairs, wherein the input is the descriptor and the output is the outcome of interest [20]. On the other hand, unsupervised ML identifies specific groups with common features within the dataset without prior knowledge of the significance of the data [20]. In 2006, AI technology had a major breakthrough with deep learning (DL), a subset of ML [21]. DL mimics the human neuronal network as it combines multiple nonlinear processing layers wherein the original data is abstracted layer-by-layer, and different levels of the abstract features are obtained and used for target detection, classification, or segmentation [21]. The primary advantage of DL over ML is that it requires minimal human intervention to generate the output of interest [21].

ANNs are a set of interconnected computers and algorithms consisting of inputs, weights, bias/threshold, and outputs that mimic human neuroanatomy [22]. However, they differ from DL due to a lower number of hidden layers within the network. In ANNs, each computing unit essentially functions as a ‘neuron’ and is connected to other computing units, building a highly complex network [20]. Through this network, signals travel to reach the output layers, traversing through multiple hidden layers [20]. As the ANNs are trained with the help of training data, the weights of the interneuron connections are adjusted to optimize output data and increase efficacy [22].

ES is a computing system capable of solving complex problems with reasoning based on current knowledge, emulating the decision-making capacity of a human expert [23]. These systems are designed to mimic clinical reasoning and judgment and have the capability to express conclusions as a probability based on input data [24]. Currently, it takes many years and a large dataset to develop a single ES capable of delivering decisions on a single output of interest or diagnosis [24]. Hence, the utilization of ES in clinical medicine is very limited. However, as AI technology continues to improve, ES may soon find widespread use in clinical medicine.

AI has found widespread application in GI, particularly for endoluminal and pancreaticobiliary disorders. It helps to significantly improve diagnostic accuracy, limit errors, standardize the interpretation of radiological and histopathological images, and establish plans for interventions [11]. Major areas of utilization of AI within GI include:

1. Application in Premalignant Lesions: Esophagogastroduodenoscopy (EGD) and colonoscopy are pivotal procedures in diagnosing upper and lower premalignant GI lesions. However, there is significant variability in premalignant lesion detection due to the endoscopists’ skill level. To standardize and improve the quality of EGDs and colonoscopies, AI-assisted models have been utilized. In current literature, two randomized controlled trials (RCTs) have compared the endoscopic performance for the diagnosis of premalignant lesions between AI-assisted and non-AI-assisted models. The WISENSE system, which used deep convolutional neural networks (CNNs) and deep reinforcement learning, reported lower rates of blind spots (5.86% vs. 22.46%, *p* < 0.001) during EGD for upper GI lesions compared to the non-AI-assisted control group [25]. The authors ultimately concluded that the WISENSE system significantly improved the quality of EGDs [25]. Another RCT by Wang et al. noted a significantly higher adenoma detection rate (ADR; 29.1% vs. 20.3%, *p* < 0.001) and mean number of adenomas per patient (0.53 vs. 0.31, *p* < 0.001) for diagnostic colonoscopy for an AI-mediated real-time automatic polyp detection system that provided audio-visual alerts upon polyp detection compared to diagnostic colonoscopies without the assistance of an AI system [26].
2. Application in Malignant Lesions: AI can help gastroenterologists accurately determine the prognosis of malignant GI neoplasms compared to conventional non-AI models [27,28,29,30]. A study by Gohari et al. compared the accuracy of prediction of survival rates for patients with colorectal cancer between an ANN AI-assisted model and Cox regression models [27]. The authors noted that the ANN model had more accurate predictions of survival for colon (89% vs. 78.6%) and rectal (82.7% vs. 70.7%) cancer patients compared to the Cox regression models [27]. Biglarian et al. compared the accuracy of prediction of distant metastasis for colorectal cancer between an ANN AI-assisted model and logistic regression models [28]. The authors observed that the ANN model had higher accuracy in predicting distant metastasis (area under the receiver operating characteristic curve (AUROC): 0.82 vs. 0.77) compared to the logistic regression models [28]. Another study by Nilsaz-Dezfouli et al. demonstrated the utility of a single time-point feed-forward ANN AI-assisted model to predict the probability of survival for gastric cancer patients at 1, 2, 3, 4, and 5 years after surgery [29]. The authors concluded that the prediction of survival for the ANN model was consistently accurate (88.7–90.2%), with sensitivity and specificity ranging from 70.2–92.5% and 66.7–96.2%, respectively [29]. Furthermore, DL algorithms have also found applications in the detection and treatment of GI malignancies [31,32,33]. A systematic review and meta-analysis of five RCTs (4354 patients) that assessed the performance of a DL computer-aided polyp detection system for the detection of colorectal neoplasia noted a significantly higher pooled adenoma detection rate (36.6% vs. 25.2%, RR 1.44; 95% confidence interval (CI) 1.27–1.62; *p* < 0.01; I2 = 42%) and adenomas detected per colonoscopy (58% vs. 36%, RR 1.70; 95% CI 1.53–1.89; *p* < 0.01; I2 = 33%) for the AI-assisted model compared to the control group [31]. From a treatment perspective, DL models can predict clinical response to chemotherapy and radiation with high accuracy (≥80%) [32,33].
3. Application in Inflammatory Lesions: Numerous studies have investigated the use of AI-assisted models to identify a wide spectrum of inflammatory lesions. For identifying patients with inflammatory bowel disease (IBD), the support vector machine (SVM) model, a type of machine learning algorithm, had diagnostic accuracy, sensitivity, and specificity ranging from 80–100%, 80–95.2%, and 92.4–93.6%, respectively, using endoscopic or wireless capsule endoscopy (WCE) images as input data [20]. The SVM model has also been used to detect ulcerative disease (peptic ulcers, ulcers from Crohn’s disease, NSAID-induced ulcers, and unexplained ulcers) with high accuracy (74–96.3%), sensitivity (75–100%), and specificity (73.3–100%) [20]. Furthermore, a study by Cui et al. used an adaptive threshold classifier AI-assisted model on 7218 small bowel WCE images to identify lymphangiectasia with a diagnostic accuracy of 97.9% [20]. Another study by Wu et al. used the Rustboost AI-assisted model on small bowel WCE images from 10 patients to identify individuals with a hookworm infection with the accuracy, sensitivity, and specificity of 78.2%, 77.2%, and 77.9%, respectively [20]. In patients with celiac disease, the diagnostic accuracy of AI-assisted models ranges from 76.7–99.6% [20].
4. Application in Gastrointestinal Bleeding: GI bleeding is a common medical emergency associated with significant morbidity and mortality. In the current literature, twelve studies have assessed the use of AI-assisted models to detect small bowel bleeding using WCE images/videos as input data [20,34,35,36,37,38,39,40,41,42,43]. Of these, six studies using an SVM AI-assisted model to identify patients with small bowel bleeding reported diagnostic accuracy ranging from 91.8–99.6% [35,36,37,39,40,41]. Additionally, five studies that utilized various AI-assisted models, such as multilayer perceptron network (MLP), probabilistic neural network, joint diagonalization principal component analysis, and CNN reported diagnostic accuracy ranging from 87.4–98% [20,34,38,42,43]. However, a study by Jung et al. that utilized a color spectrum transformation AI-assisted model to identify small bowel GI bleeding using WCE images as input data had a diagnostic accuracy of only 30% but a sensitivity and specificity of 94.9% and 96.1%, respectively [20].
5. Application in Hepatology: The utilization of AI-assisted models to detect liver fibrosis, non-alcoholic fatty liver disease (NAFLD), and esophageal varices has increased exponentially in recent years. Seven studies that used AI-assisted models to detect liver fibrosis associated with viral hepatitis (hepatitis B and C viruses) reported diagnostic accuracy of ≥84.4% [20]. The diagnostic accuracy of AI-assisted models from six studies that aimed to identify individuals with NAFLD ranged from 79% to 89% [20]. Two studies that used MLP and random forest AI-assisted models to detect esophageal varices noted a diagnostic accuracy of 87.8% and 0.82 (AUROC), respectively [20]. Overall, these AI models identified their target factor with ≥80% accuracy.

### 2.2. Utilization of Artificial Intelligence in Endoscopic Ultrasound for the Detection of Pancreatic Cancer

In the US, the incidence and prevalence of pancreatic cancer continue to rise [6]. It is currently the third leading cause of cancer mortality and soon will be the second, behind lung cancer [10]. Despite these rising trends, there are no definitive guidelines on pancreatic cancer screening in average-risk individuals. Imaging modalities such as CT scans and MRIs are often used to aid the diagnosis of pancreatic cancer, but EUS is considered far superior due to its higher diagnostic yield and ability to obtain high-quality images [44]. However, there are some limitations to conventional EUS, such as low sensitivity in differentiating benign from malignant intraductal papillary mucinous neoplasms (IPMNs) and low specificity in differentiating chronic pancreatitis (CP) from malignant pancreatic lesions [44,45,46]. Furthermore, EUS is highly operator-dependent, and therefore, less experienced endoscopists may not be able to appreciate the subtle differences between CP and pancreatic cancer due to the presence of concomitant scarring and calcification secondary to the presence of chronic inflammation [44,45,46].

Numerous studies have been performed to assess and compare the diagnostic accuracy of non-AI and AI-augmented models of EUS for pancreatic cancer (Table 1). A retrospective study of 50 patients with IPMN, which used EUS images as input data for a DL algorithm, reported the sensitivity, specificity, and accuracy of 95.7%, 92.6%, and 94.0%, respectively, for malignant IPMNs [47]. This far exceeded the accuracy of human diagnosis [56.0%] [47]. Another retrospective study by Zhang et al. utilized SVM for EUS images from 216 patients to assess the ability of the SVM AI-assisted model to differentiate normal tissue from pancreatic cancer [48]. All 216 of these patients underwent EUS-guided fine-needle aspiration (EUS-FNA) and pathologic analysis to correlate findings with the definitive diagnosis [48]. The authors concluded that the SVM model had the accuracy, sensitivity, and specificity of 98%, 94.3%, and 99.5%, respectively [48]. Therefore, it could be used as a rapid, non-invasive test for pancreatic cancer screening [48]. Ozkan et al. conducted a retrospective study to develop a high-performance computer-aided diagnosis (CAD) system with image processing and pattern recognition abilities using ANNs [49]. The input data for the ANN was collected from EUS images of 332 patients. which were classified into three groups based on patient age (<40, 40–60, and >60 years old) [49]. The authors observed that the CAD system performed significantly better, with a sensitivity of 83.3%, specificity of 93.3%, and diagnostic accuracy of 87.5% when the images were classified according to the patient’s age, reflecting the importance of age in aiding the diagnosis of pancreatic cancer [49]. Furthermore, in a systematic review of 11 studies examining the role of AI-assisted EUS models in diagnosing pancreatic cancer, the overall accuracy, sensitivity, and specificity were found in the ranges of 80–97.5%, 83–100%, and 50–99%, respectively [50]. Based on current data, AI-assisted EUS models have great potential as diagnostic tools for detecting pancreatic cancer.

### 2.3. Utilization of Artificial Intelligence in Endoscopic Ultrasound to Differentiate Pancreatic Cancer from Chronic Pancreatitis

Over the last decade, imaging modalities for pancreatic lesions have improved significantly. However, differentiating between PDAC and CP is a diagnostic challenge as CP often mimics the radiological features of PDAC [51]. Cytological analysis continues to be the gold-standard test to differentiate PDAC from CP. Additionally, CP is a risk factor implicated in the development of PDAC. Hence, both clinical entities may co-exist together in the same patient [52]. In these complex cases, AI-assisted diagnostic models may help establish an accurate diagnosis. A retrospective study conducted by Das et al. for 56 patients using EUS images for digital image analysis (DIA) by an ANN noted that the AI-assisted model was highly accurate in differentiating between normal tissue, CP, and PDAC (area under the curve (AUC) of 0.93 for PDAC) [53]. Even in experienced hands, EUS imaging alone may require supplementation with FNB to differentiate malignancy from CP. Another retrospective analysis compared the accuracy of differentiation of pancreatic cancer from focal pancreatitis by an endosonographer versus a self-learning ANN model using EUS images as input data for 21 pancreatic cancer and 14 focal pancreatitis patients [54]. The authors reported that the maximal accuracy of the AI-assisted software (89%) compared favorably with the accuracy of human interpretation (85%) [54]. A cross-sectional study of 68 patients (32 PDAC, 22 normal pancreas, 11 CP, and 3 pancreatic neuroendocrine tumors) to assess the accuracy of extended neural network (ENN)-assisted real-time EUS elastography yielded an average testing performance of 95% and a high training performance of 97% in differentiating benign and malignant masses [55]. Tonozuka et al. used EUS images to develop a computer-assisted diagnosis (CAD) system using a DL model and evaluated its ability to detect PDAC using control EUS images from CP and normal pancreas patients [56]. The EUS-CAD model demonstrated excellent results (AUC 0.924 and 0.940 in the validation and test settings, respectively) in detecting PDAC [56]. Furthermore, a study by Zhu et al. utilized EUS image parameters for an SVM predictive model to differentiate pancreatic cancer and CP for 388 patients (262 pancreatic cancer and 126 CP) [57]. The authors reported the average accuracy, sensitivity, and specificity of 94.2%, 96.3%, and 93.4%, respectively, for the SVM predictive model [57].

Although numerous studies have demonstrated high diagnostic accuracy and strongly encourage the use of AI-assisted models to differentiate PDAC from other benign lesions, the main drawback is the small patient population used in each analysis, which significantly limits the input data for these AI-assisted models. Hence, multicenter studies were conducted to further validate these findings. A prospective multicenter-blinded analysis using ANN-assisted real-time EUS elastography was conducted for 258 patients at 13 tertiary academic medical centers in Europe to differentiate between pancreatic cancer and CP [58]. The authors observed that the AI-assisted model had a 91.1% training accuracy (95% CI: 89.87%–92.42%) and an 84.3% testing accuracy (95% CI, 83.09–85.44%), implying that the use of ANNs provided fast and accurate diagnoses for pancreatic malignancies [58]. Another observational prospective multicenter study that included 167 consecutive patients (112 pancreatic cancer and 55 CP) from Romania, Denmark, Germany, and Spain used parameters from the time-intensity curve (TIC) analysis of contrast EUS in an ANN model to differentiate pancreatic cancer and CP [59]. The authors reported that ANNs had high sensitivity (94.64%), specificity (94.44%), positive predictive value (97.24%), and negative predictive value (89.47%) and could be used to differentiate pancreatic cancer from CP with a high degree of accuracy [59]. 

In conclusion, all studies—large or small—have concluded that AI-assisted EUS models can be used in clinical practice to differentiate pancreatic cancers from CP with excellent results (Table 2).

### 2.4. Utilization of Artificial Intelligence in Endoscopic Ultrasound to Differentiate Pancreatic Cancer from Autoimmune Pancreatitis

AIP has been recognized as a distinct and rare fibroinflammatory subtype of chronic pancreatitis. It has characteristic features on sonographic and cross-sectional radiological imaging that mimic PDAC [60,61]. This may lead to a delayed or incorrect diagnosis. AI-assisted models can help solve this diagnostic dilemma. Mayra et al. conducted a study using a database of still images and video data from EUS examinations of 538 patients in the US to develop an EUS-based CNN model that can differentiate AIP from PDAC [62]. The authors reported that the EUS-based CNN model was 90% sensitive and 93% specific in distinguishing AIP from PDAC [62]. These findings encourage the use of AI-assisted EUS models in these subset patients for an early and accurate diagnosis. However, the data on AI-assisted EUS models to distinguish PDAC from AIP is still limited and warrant additional large multicenter prospective studies. 

### 2.5. Limitations of Artificial Intelligence in Endoscopic Ultrasound for the Detection of Pancreatic Cancer

Even at the current early stage of development, AI-assisted diagnostic models provide significant value in aiding medical decision-making and planning therapeutic interventions for patients with pancreatic cancer. However, there continues to be hesitancy in their application in clinical practice by most practitioners despite promising results. In recent years, more studies reporting a higher diagnostic accuracy of AI-assisted EUS models compared to human interpretation for pancreatic cancer continued to be published. These studies are slowly changing the current landscape and building confidence in AI-assisted diagnostic models as an indispensable tool in modern medicine [50,63]. However, like any diagnostic test, AI-assisted EUS models have their own set of limitations, which will need to be addressed before they can be used as a ‘go-to’ diagnostic test for pancreatic cancer.

One of the most important limitations of an AI-assisted EUS model is the lack of adequate standardization of input data that are used to train the AI algorithm [63]. As per current literature, no standardized protocols for data collection, processing, and storage for the AI-assisted model have been established. Additionally, standardized principles for data analysis by the AI algorithm are also lacking. Establishing these protocols is important because if the AI-assisted EUS model trains on data that are misrepresentative of PDAC population variability, it is likely to reinforce bias, which may lead to inaccurate diagnoses, lack of generalizability, and, ultimately, adverse patient outcomes [64]. Furthermore, different types of AI-assisted EUS models may require images of the area of interest prepared in a specific manner and may not perform with a high degree of accuracy with different imaging subsets. Although universal protocols can be created for input data to increase the efficiency and accuracy of AI-assisted EUS models, it may be an extremely time and labor-expensive process [63].

Another area of concern is the quality of input data used to train the AI-assisted EUS models. Most studies in the current literature derive input data from a single institution, with only a few multicenter experiences [50,58,59,63]. This lack of diversity in the dataset leads to an information bias. For the AI-assisted EUS diagnostic models to achieve a high degree of diagnostic accuracy and generalizability to diagnose and differentiate PDAC from other etiologies, the dataset needs to be highly diverse, capturing all possible variations and variables used in the decision-making process [63]. This can be achieved by developing a quality-monitored central data collection server for EUS images from all institutions across the US, both academic and private. Furthermore, just collecting high-quality data is not sufficient. It is also imperative to ensure that the studies that utilize the data to report specific outcomes on pancreatic cancers must have high methodological quality and standards of reporting as they may influence current guidelines or help in developing future ones [65]. Poor quality studies with flawed methodologies and a lack of transparent reporting may create distrust among healthcare professionals, leading to delays in policy changes and the adoption of this newer technology in current clinical practice [65]. 

The AI ‘Black Box’ problem, particularly for ML and DL AI-assisted models, has garnered significant attention and is rapidly becoming a concern [66]. A ‘Black Box’ AI is an AI algorithm that allows the observer to visualize input and output data without any information on the processes and operations used to derive the output data [66,67]. Hence, the observer is unable to interpret and determine the reasoning behind how a specific variable was weighed within the AI algorithm [63,66,67]. This is concerning as gastroenterologists need to be able to visualize and understand how the information on PDAC was processed and analyzed to prevent errors that can ultimately lead to adverse patient outcomes. Therefore, for the time being, AI-assisted EUS models should be used as adjuncts to clinical experience rather than ultimate answers when recommending treatment for pancreatic cancers. 

**Figure 2:**
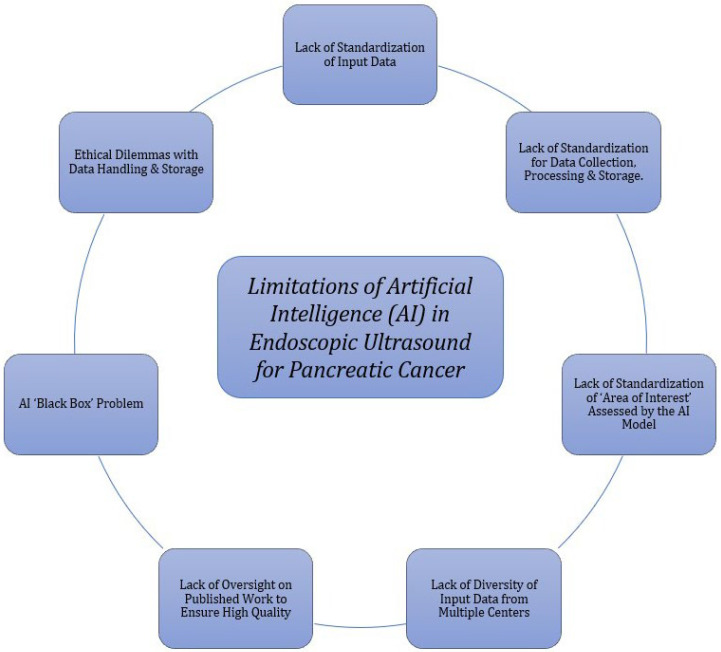
Limitations of artificial intelligence in endoscopic ultrasound for pancreatic cancer.

Finally, there are numerous ethical dilemmas associated with the handling and storage of sensitive patient information [68]. As the AI-assisted EUS models require a great volume of input data, appropriate de-identification of patient information is required to protect patient privacy, reduce bias, and ensure the fairness of the algorithm [68]. However, the de-identified data needs to be traceable back to the patient to aid in diagnosis and recommend treatment options. Furthermore, this information needs to be secure from cybercriminals and interested parties who may use it to exploit vulnerabilities and influence the healthcare of these individuals [68]. Figure 2 summarizes all the limitations associated with AI in EUS. 

### 2.6. Future Directions of Artificial Intelligence in Endoscopic Ultrasound for Pancreatic Cancer

Despite its limitations, the growth and application of AI in different subspecialties of medicine, particularly GI, have increased exponentially. Collaborations between academic centers, private physicians, and industry will continue to drive the AI revolution to improve its quality, utility, ease of use in everyday clinical settings, and, most importantly, accuracy for the early detection of pancreatic cancer. We foresee the following ‘near’ and ‘far’ future applications of AI in EUS for patients with pancreatic cancer:

#### 2.6.1. ‘Near’ Future Application of Artificial Intelligence in Endoscopic Ultrasound for Pancreatic Cancer

**Figure 3:**
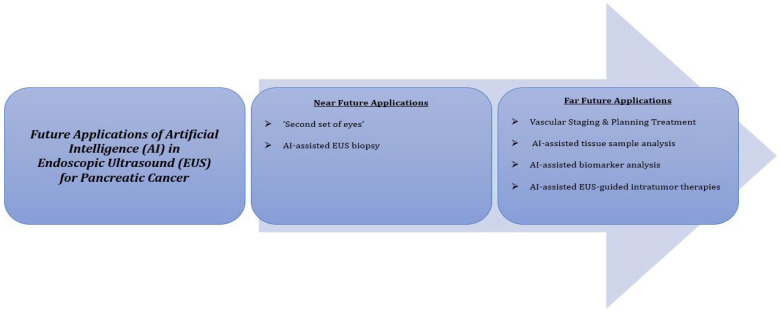
Future applications of artificial intelligence in endoscopic ultrasound for pancreatic cancer.

The diagnostic accuracy of AI-assisted EUS models has been compared favorably to or exceeded the diagnostic accuracy of human interpretation for pancreatic cancers [47,54]. However, it is worth noting that neither AI-assisted models nor human diagnosis has 100% diagnostic accuracy. Hence, we strongly believe that AI-assisted models should serve as a ‘second set of eyes’ to the endosonographer rather than a replacement (Figure 3). Furthermore, AI-assisted models can also potentially aid experienced endosonographers during biopsies while, at the same time, learning from these experts in the field. These strategies may be critical in the early detection of pancreatic cancer and differentiating them from other clinical entities. Ultimately, AI-assisted EUS models should help reduce operator variability, which has been a traditional limitation of EUS.

#### 2.6.2. ‘Far’ Future Application of Artificial Intelligence in Endoscopic Ultrasound for Pancreatic Cancer

The application of AI technology in clinical medicine is still in the preliminary phase, with a wide scope for improvement and utilization. As AI-assisted models are ideal for the analysis of large datasets, they will have widespread utility in composite imaging, which includes the fusion of EUS and cross-sectional radiological imaging to determine vascular staging for pancreatic cancers. This information will be vital to endoscopists and other specialties (radiology, medical oncology, surgical oncology, and radiation oncology) involved in all aspects of pancreatic cancer staging. Additionally, it will help plan appropriate interventions and recommend treatment options.

AI-assisted models analyzing samples from EUS-FNA and EUS-FNB are also quickly gaining traction due to their ability to differentiate complex tissue specimens [69]. The diagnostic accuracy of MLP EUS-FNA and CNN EUS-FNB models for differentiating pancreatic cancer from other pancreatic tumors was reported to be 100% and 94.17%, respectively. However, as AI technology advances, with better AI algorithms and improved quality of EUS images/videos as input data, AI-assisted EUS models may replace traditional EUS-FNA/FNB as the gold-standard test for diagnosing pancreatic cancer due to their less invasive nature and high diagnostic accuracy. 

Cancer biomarkers have widespread utility in screening, differential diagnosis, staging, risk assessment, response to treatment, monitoring disease progression, and the prognosis of any cancer [70]. However, biomarkers for pancreatic cancer currently lack sufficient sensitivity and specificity for widespread clinical application. Hence, this is an area of interest where AI-assisted models may be highly beneficial. Large sets of biomarker data from EUS-guided liquid biopsies can be analyzed using AI technology to identify pancreatic cancer at an early stage [69]. Studies investigating AI algorithms capable of analyzing biomarker data with high accuracy are an area of active research. 

From an intervention perspective, AI-assisted EUS-guided fine-needle injection is another potential area of application of AI technology. Using AI-assisted real-time EUS imaging guidance, interventional endoscopists may be able to directly inject activated allogeneic lymphocyte culture or oncolytic attenuated adenovirus (ONYX-015), which are currently being studied as a potential therapy for pancreatic cancer, directly into pancreatic lesions. Traditionally, pancreatic lesions have been extremely difficult to reach by a percutaneous approach due to their depth. Hence, AI-assisted EUS-guided fine-needle injection may become the preferred approach in elderly patients or those with a high comorbidity burden, primarily due to the less invasive nature of the procedure and fewer complications. However, additional research is needed before AI-assisted EUS-guided fine-needle injection finds widespread application. Figure 3 summarizes all the potential future applications of AI in EUS for pancreatic cancer.

## 3. Conclusions

AI-assisted EUS models have shown promise in the early detection of pancreatic cancer, with a high degree of accuracy despite still being in the infancy of development and utilization. Compared to human interpretation, AI technology has either been compared favorably or has been noted to be far superior in identifying pancreatic cancer and differentiating it from other clinical entities that mimic pancreatic cancer on conventional radiological imaging such as CP and AIP. However, AI-assisted EUS models have a unique yet concerning set of limitations, such as the AI ‘Black Box’, the lack of adequate standardization and quality of data, and ethical dilemmas associated with sensitive patient information, all of which limit widespread application. Despite these limitations, AI technology may be instrumental in transforming the future of healthcare, especially for pancreatic cancer, due to its precision in analyzing and processing large datasets. AI-assisted EUS models could serve as a ‘second set of eyes’ to the endosonographer, improving diagnostic accuracy. AI technology could also assist in composite imagining to determine the vascular staging for pancreatic cancers and in AI-assisted EUS-guided fine-needle injection to easily treat deep pancreatic lesions. Most studies on AI are retrospective; hence, large-scale prospective clinical trials are needed to accurately evaluate the diagnostic accuracy of AI algorithms in real-world clinical settings. If successful, AI-assisted EUS models have the potential to become an indispensable tool in the management of patients with pancreatic cancer. 

## Figures and Tables

**Table 1:** Studies assessing the sensitivity, specificity, and diagnostic accuracy of artificial intelligence (AI)-augmented and non-AI models for pancreatic cancer.

Study	Study Design	Artificial Intelligence Model	Patient Population	Outcomes for the Artificial Intelligence Model
Kuwahara et al. [47]	Retrospective (Japan)	Deep Learning (Convolutional Neural Networks (CNNs))	Total IPMN Patients = 50 Benign IPMN Patients = 27 Malignant IPMN Patients = 23	Recognition of Malignant IPMN: Sensitivity = 95.7% Specificity = 92.6% Accuracy = 94%
Zhang et al. [48]	Retrospective (China)	Support Vector Machine (SVM)	Total Patients = 216 Pancreatic Cancer Patients = 153 Non-Cancer Patients = 63	Recognition of Pancreatic Cancer: Sensitivity = 94.32% Specificity = 99.45% Accuracy = 97.98%
Ozkan et al. [49]	Retrospective (Turkey)	Artificial Neuronal Networks (ANNs)	Total Patients = 332 Pancreatic Cancer Patients = 202 Non-Cancer Patients = 130	Recognition of Pancreatic Cancer (All Ages): Sensitivity = 83.3% Specificity = 93.33% Accuracy = 87.5% Recognition of Pancreatic Cancer (>60 years): Sensitivity = 93.3% Specificity = 88.88% Accuracy = 91.66% Recognition of Pancreatic Cancer (40–60 years): Sensitivity = 85.7% Specificity = 91.66% Accuracy = 88.46% Recognition of Pancreatic Cancer (<40 years): Sensitivity = 87.5% Specificity = 94.11% Accuracy = 92%
Goyal et al. [50]	Systematic Review	Artificial Neural Network (ANN) Convolutional Neural Networks (CNNs) Support Vector Machine (SVM)	Total Patients = 2292 Pancreatic Cancer Patients = 1409 Non-Cancer Patients = 883	Recognition of Pancreatic Cancer: Sensitivity = 83–100% Specificity = 50–99%, Accuracy = 80–97.5%

IPMN: intraductal papillary mucinous neoplasm.

**Table 2:** Studies comparing artificial intelligence (AI)-augmented models to differentiate pancreatic cancer from other clinical entities.

Study	Study Design	Artificial Intelligence Model	Patient Population	Outcomes for the Artificial Intelligence Model
Das et al. [53]	Retrospective (United States)	Artificial Neural Network (ANN)	Normal Pancreas Patients = 22 Chronic Pancreatitis Patients = 12 Pancreatic Cancer Patients = 22	Recognition of Pancreatic Cancer: Sensitivity = 93% Specificity = 92% Recognition of Chronic Pancreatitis versus Normal Pancreas: Sensitivity = 100% Specificity = 100%
Norton et al. [54]	Retrospective (United States)	Artificial Neural Network (ANN)	Total Patients = 35 Pancreatic Cancer Patients = 21 Focal Pancreatitis Patients = 14	Recognition of Pancreatic Cancer by AI: Sensitivity = 100% Specificity = 50% Accuracy = 80% Recognition of Pancreatic Cancer by EUS: Sensitivity = 89% Specificity = 79% Accuracy = 85% Recognition of Pancreatic Cancer by Human Interpretation: Sensitivity = 73% Specificity = 100% Accuracy = 83%
Săftoiu et al. [55]	Retrospective (Europe)	Artificial Neural Network (ANN)	Total Patients = 68 Pancreatic Cancer Patients = 32 Pancreatic Neuroendocrine Tumor Patients = 3 Chronic Pancreatitis Patients = 11 Normal Pancreas Patients = 22	Recognition of Pancreatic Cancer and Pancreatic Neuroendocrine Tumors: Sensitivity = 91.4% Specificity = 87.9% Accuracy = 89.7%
Tonozuka et al. [56]	Cross-Sectional (Japan)	Convolutional Neural Networks (CNNs)	Total Patients = 139 Pancreatic Cancer Patients = 76 Chronic Pancreatitis Patients = 34 Normal Pancreas Patients = 29	Recognition of Pancreatic Cancer (Validation Set): Sensitivity = 90.2% Specificity = 74.9% Area Under the Curve = 0.924 Recognition of Pancreatic Cancer (Test Set): Sensitivity = 92.4% Specificity = 84.1% Area Under the Curve = 0.940
Zhu et al. [57]	Retrospective (China)	Support Vector Machine (SVM)	Total Patients = 388 Pancreatic Cancer Patients = 262 Chronic Pancreatitis Patients = 126	Recognition of Pancreatic Cancer: Sensitivity = 96.25% Specificity = 93.38% Accuracy = 94.2%
Săftoiu et al. [58]	Prospective Multicenter (Europe)	Artificial Neural Network (ANN)	Total Patients = 258 Pancreatic Cancer Patients = 211 Chronic Pancreatitis Patients = 47	Recognition of Pancreatic Cancer: Sensitivity = 87.59% Specificity = 82.94% Area Under the Curve = 0.94
Săftoiu et al. [59]	Prospective Multicenter Observational (Europe)	Artificial Neural Network (ANN)	Total Patients = 167 Pancreatic Cancer Patients = 112 Chronic Pancreatitis Patients = 55	Recognition of Pancreatic Cancer by AI: Sensitivity = 94.64% Specificity = 94.44% Recognition of Pancreatic Cancer by Contrast-Enhanced EUS: Sensitivity = 87.5% Specificity = 92.72%

AI: artificial intelligence. EUS: endoscopic ultrasound.

## Data Availability

All data utilized for this narrative review is publicly available on PubMed and embedded in the reference section of the manuscript.
